# Supplementary material for: Fibrillarin Ribonuclease Activity is Dependent on the GAR Domain and Modulated by Phospholipids
Source: Cells. 2020 May 6;9(5):1143. doi: 10.3390/cells9051143 (PMC7290794; doi:10.3390/cells9051143)
Supplement: Supplementary file 1 [file cells-09-01143-s001.pdf]

Supplementary figures

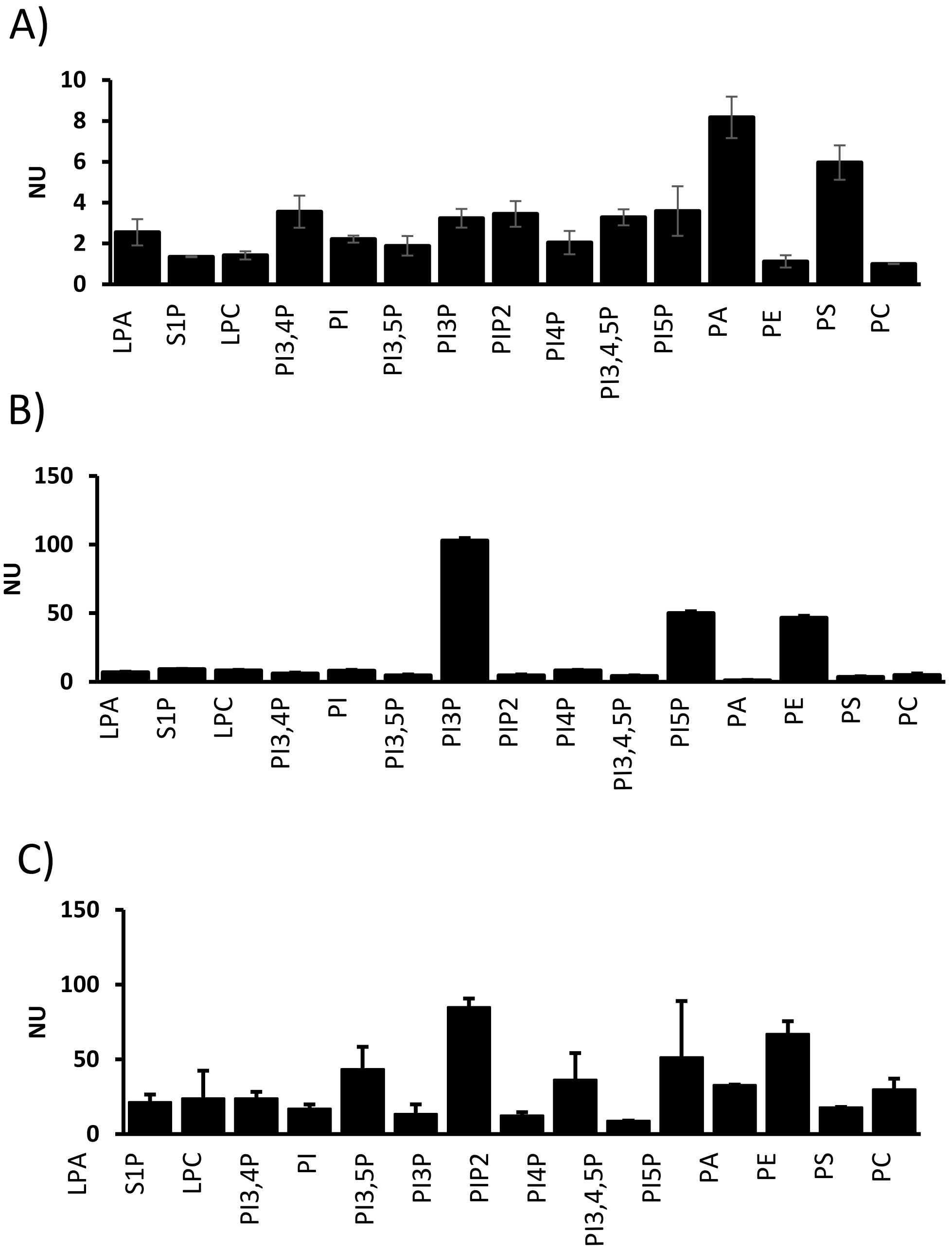

Supplementary figure 1. Quantification of Fat blot signals. Signal quantification from figures 1E and 5C were made by ImageJ software. The bars indicate the average and standard deviation (n = 2). Fat blot densitometry analysis from A) correspond to WT fibrillarin, B) for R34A mutant and C) for R45A mutant

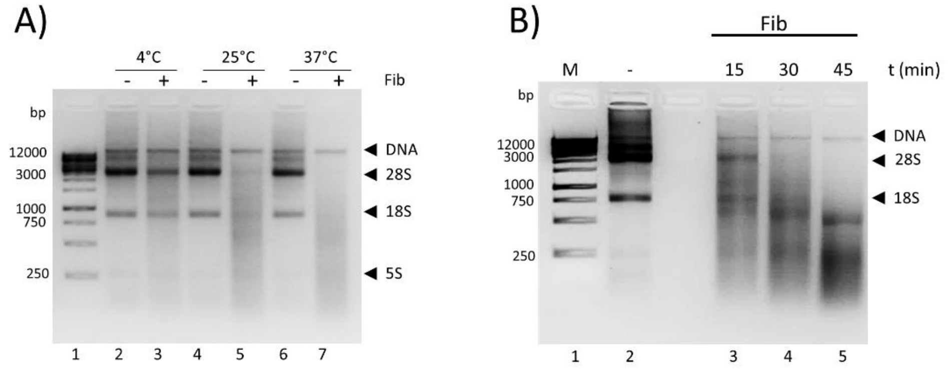

Supplementary figure 2. Evaluation of the ribonuclease activity of fibrillarin. The rRNA degradation activity of fibrillarin was tested for 45 min at three different temperatures (4, 25 and 37°C) (A). It is noted that at 4°C the rRNA degradation is minimal with only one 3000 pb RNA fragment degraded (lane 3). At 25 and 37°C additional degradation of the rRNA was observed (lanes 5 and 7). Of note, DNA derived from RNA extraction remains intact. Time dependent activity was also evaluated at 15, 30 and 45 min at 37 °C (B).

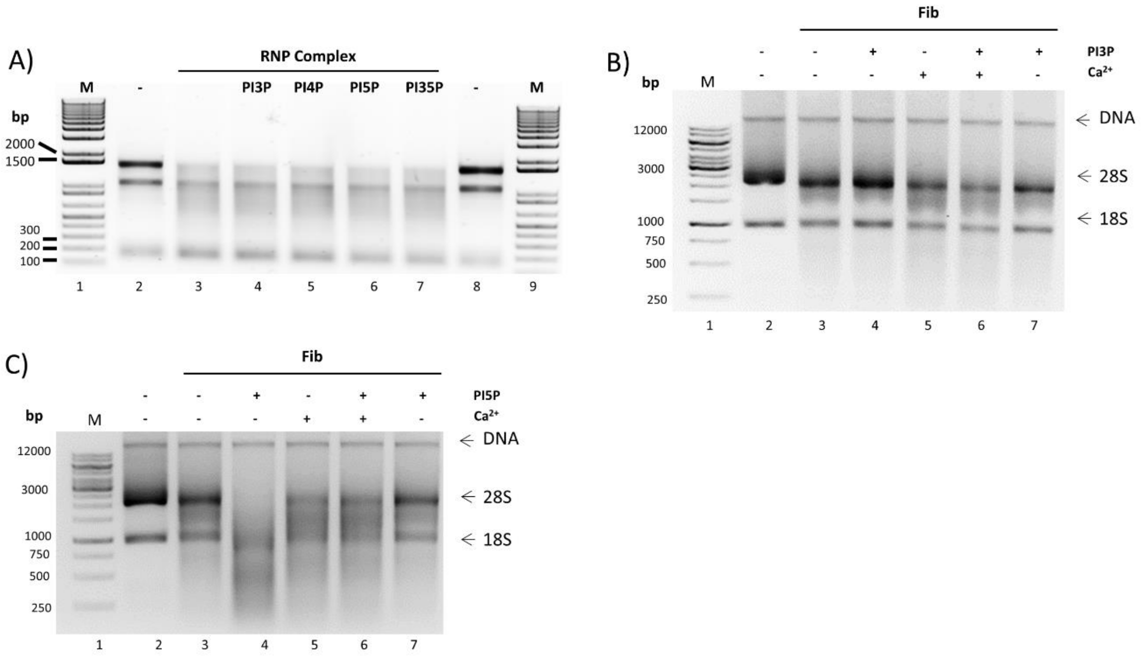

Supplementary figure 3. Ribonuclease activity assay of fibrillarin in the RNP complex or fibrillarin alone in the presence of different phosphoinositides. The activity of RNP complex was evaluated for 45 min at 37°C in presence of PIP3, PI4P, PI5P, and PI35P at a final amount of 5 ng (A). The ribonuclease activity of fibrillarin alone was measured for 45 min at 37 °C in the presence of (B) PI3P and (C) PI5P.

A)

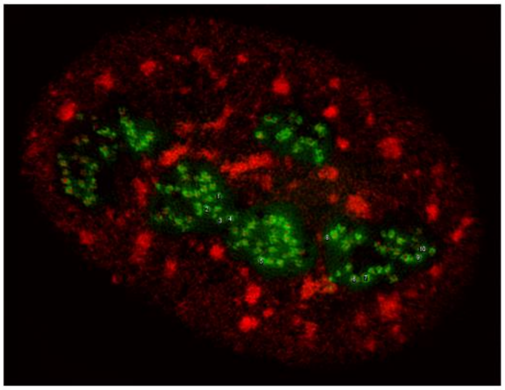

B)

PIP2	Bead diameter (µm)	Fibrillarin	Bead diameter (µm)
1	0.161	1	0.517
2	0.141	2	0.43
3	0.17	3	0.427
4	0.228	4	0.513
5	0.206	5	0.472
6	0.26	6	0.635
7	0.207	7	0.552
8	0.181	8	0.412
9	0.161	9	0.416
10	0.17	10	0.513
average	0.1885	average	0.4887

C)

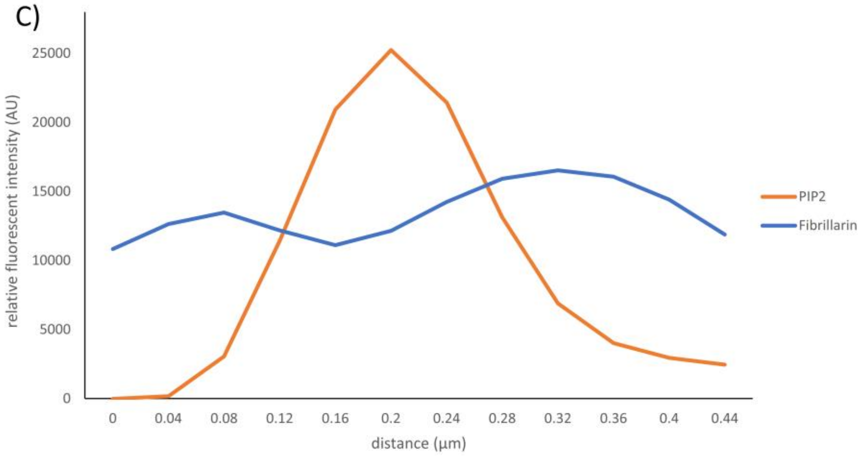

Supplementary figure 4. A) SIM image of SNAP FIB and Immunolocalize PIP2. ROI line were drawn across the fibrillarin rings and quantified as shown in B and Graph according to the intensity of the pixel

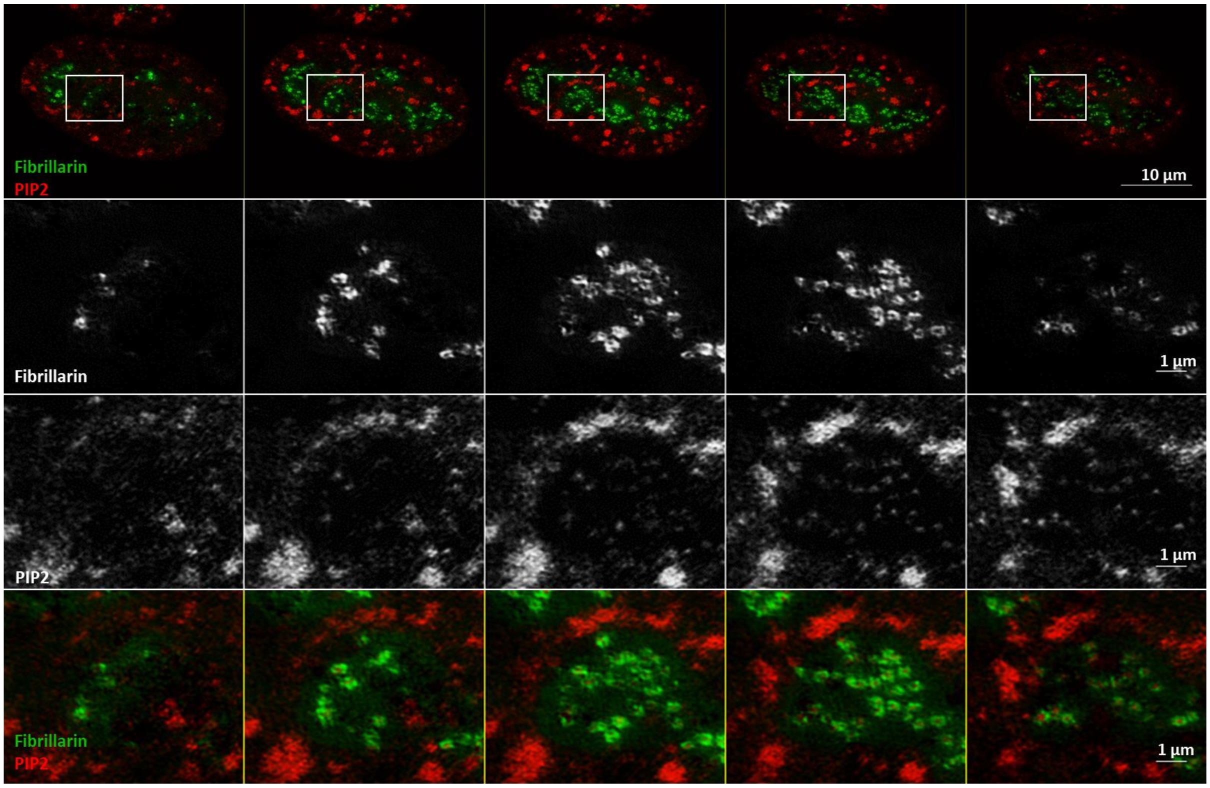

Supplementary figure 5. SIM images taken along Z stack with 0.125µM. SNAP-fibrillarin is in green and anti PIP2 is shown in red. The inset from the first row of figures was magnified and showed each channel or merged as pointed in the figure.

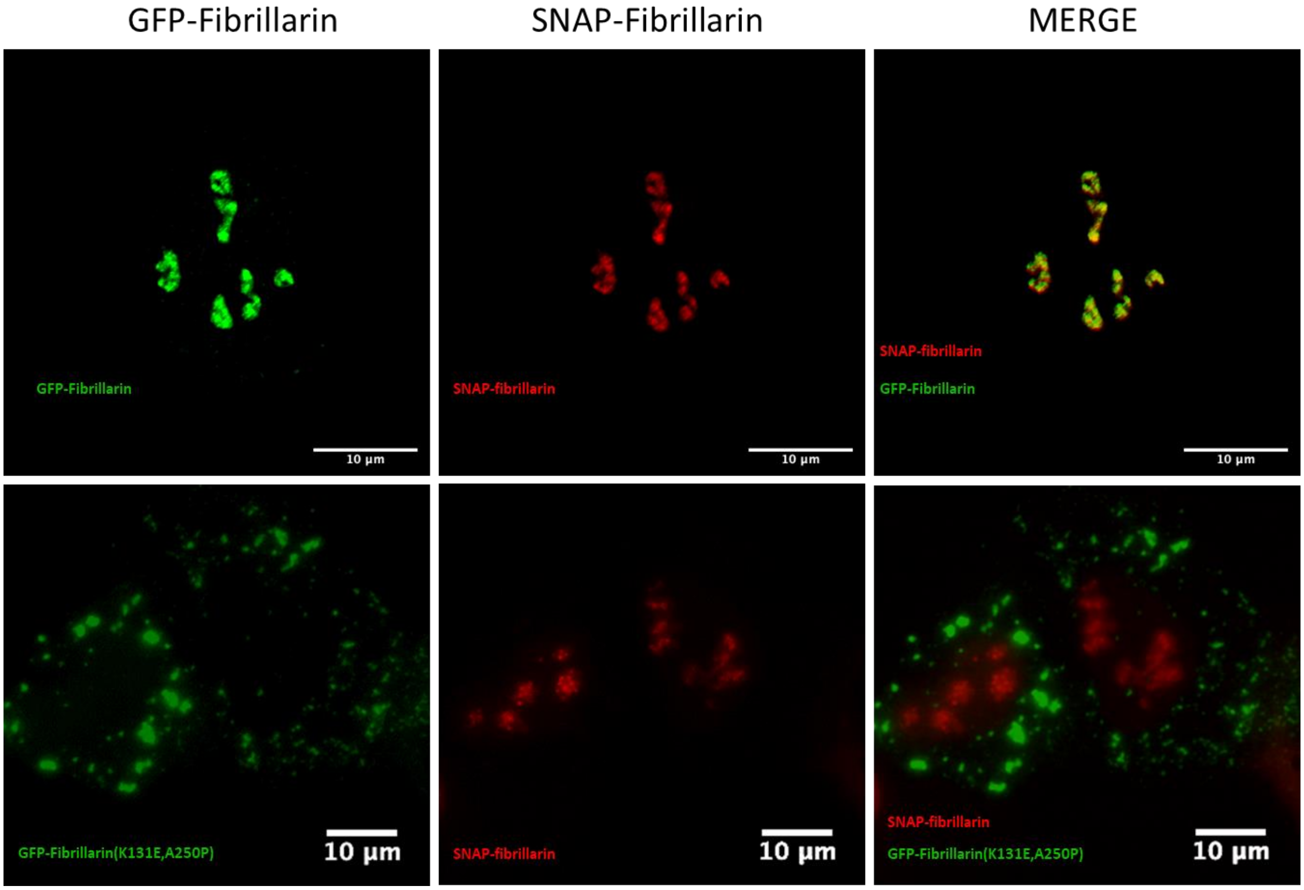

Supplementary figure 6. Co-localization of GFP and SNAP tagged Fibrillarin. Stable cell culture expressing SNAP-Fibrillarin (red). The stable cell line was transient transfected with wild type GFP-Fibrillarin (green) on top and mutant GFP-Fibrillarin (K131E, A250P) in the lower row.

Fibrillarin mutagenesis

Mutagenesis was performed with Phusion Hot Start II DNA Polymerase and the PCR product processed DpnI, kinase and ligase enzymes using specific primers for each mutation. The vector pGFP-Fibrillarin was used as DNA template for the first mutagenesis cycle and confirmed by sequencing. For the second mutation, the sequence confirmed of mutant K131E was used as template for the A250P mutation. Primers used for K131E mutant are the following: Fw 5'-AGGAGATGACGAAATTGAGTACC-3' and Rv 5'-TCCGAAATCGAGACTCTC-3'. Their transient transfection in Fibrillarin-SNAP stable cell line was performed at 80% confluence using polyethylenimine (PEI) with 10 µg of fibrillarin-mutant K131E, A250P-GFP plasmid 1 ml of DMEM (Dulbecco's Modified Eagle's Medium) without fetal calf serum. Transfection cocktail were vortexed and incubated 5 min at RT, added dropwise to the cell culture. The fluorescent substrate used for this work was SNAP-Cell® TMR-Star (555 nm) is a red fluorescent substrate added by dropwise in the culture media and washed after 5 min to stain the fibrillarin with SNAP tag.After 48 hr of incubation after transient transfection the TMR-Star (555 nm) was added and the cells were fixed in 4% with formaldehyde solution in PBS for 15 min and washed once in PBS and analyzed by microscopy. Images were acquired with a DM6000B fluorescent microscope, the illumination with Leica EL6000 with HXP 120W / 45C VIS Hg lamp for fluorescent lamp for transmitted light. Using a 555nm channel for SNAP tag, 488 for GFP tag.

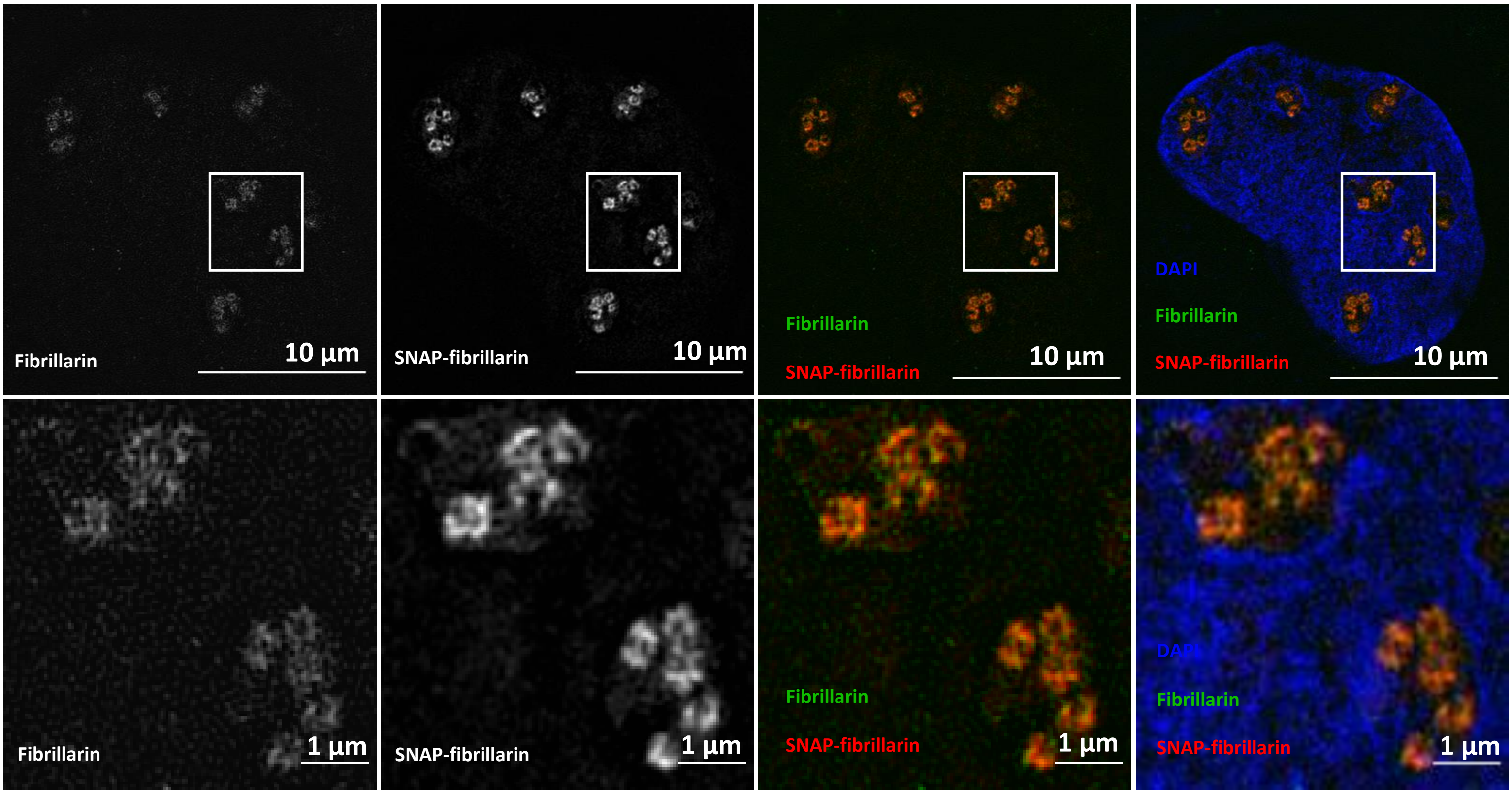

Supplementary figure 7. Co-localization of immunolocalization fibrillarin and SNAP tagged Fibrillarin. Stable cell culture expressing SNAP-Fibrillarin (red) and immunolocalize fibrillarin in green. Black and white slides only show a specific channel. 488 nm for anti-fibrillarin and 555 nm for the SNAP-Fibrillarin. The Inset shows a typical nucleolus of the cell

Methodology for figure 7

Snap-fibrillarin cells were feed for half an hour with SNAP-Cell® TMR-Star then wash with PBS, fixed, permeabilized and immunotreated as previously publish (25). We used rabbit polyclonal anti-Fib antibody (H-140, Santa Cruz Biotechnology, Dallas, TX, USA), secondary anti-rabbit polyclonal IgG Cat # A-21206 Alexa Fluor® 488, Invitrogen

Structured illumination microscopy images were acquired using the 3D-SIM system DeltaVision OMX (GE Healthcare Life Sciences, Marlborough, MA, USA) with PLAN APO N 60x/1.42 OIL objective, 4x pco.edge 5.5 sCMOS camera and DeltaVision OMX (Buckinghamshire, UK) controlling software. Lasers with wavelengths 405 nm, 488 nm, 568 nm, and 642 nm with filters for DAPI (Ex: 395.5/29; Em: 435.5/31), FITC (Ex: 477/32; Em: 528/48), Alexa Fluor 568 (Ex: 571/19; Em: 609/37). Immersion oil laser liquid (Cargille Laboratories, Cedar Grove, NJ, USA; Code: 5610; n = 1.5160). Image reconstruction and registration was processed with DeltaVision softWoRx 6.5.2. Software. Wiener filters were set to 0.001. Samples were mounted to 90% glycerol -supplemented with 5% N-propyl gallate.

A

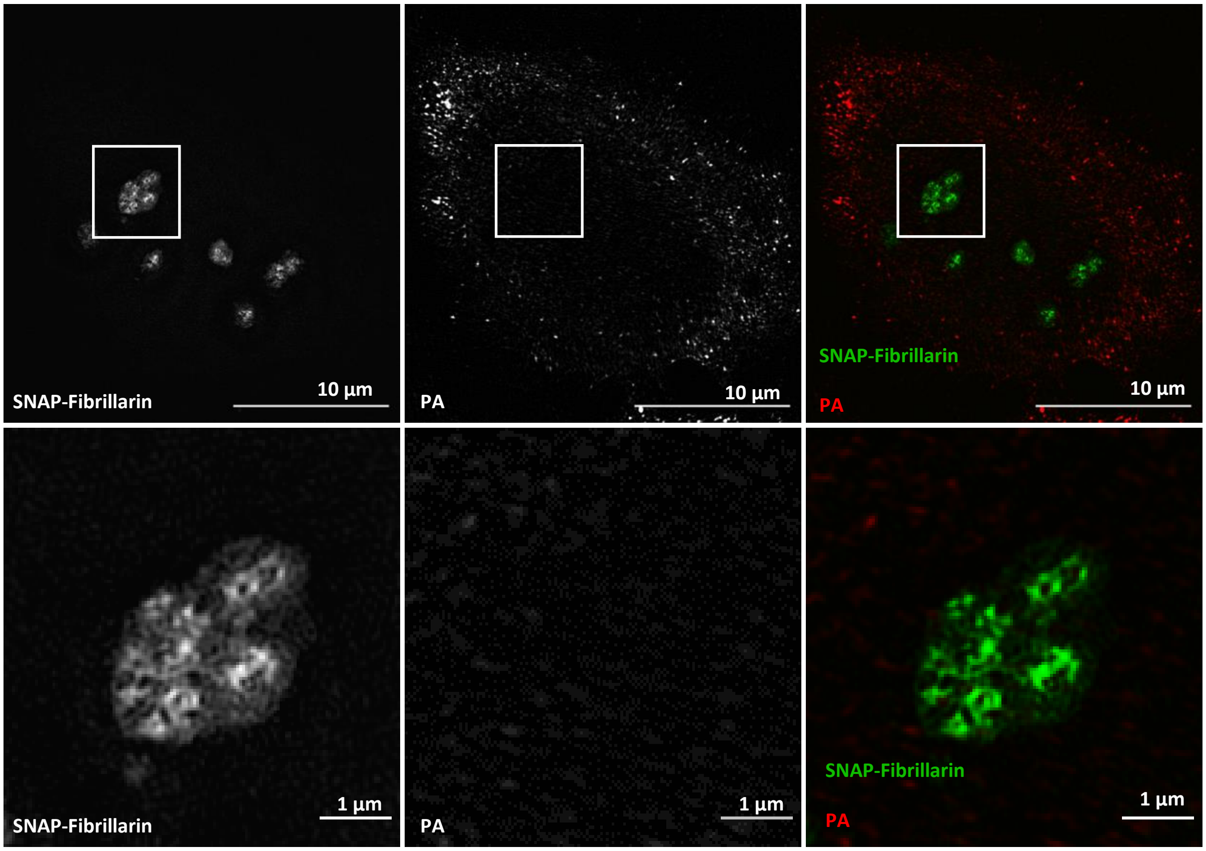
SNAP-Fibrillarin
10 µm
PA
10 µm
SNAP-Fibrillarin
PA
10 µm
SNAP-Fibrillarin
1 µm
PA
1 µm
SNAP-Fibrillarin
PA
1 µm

B

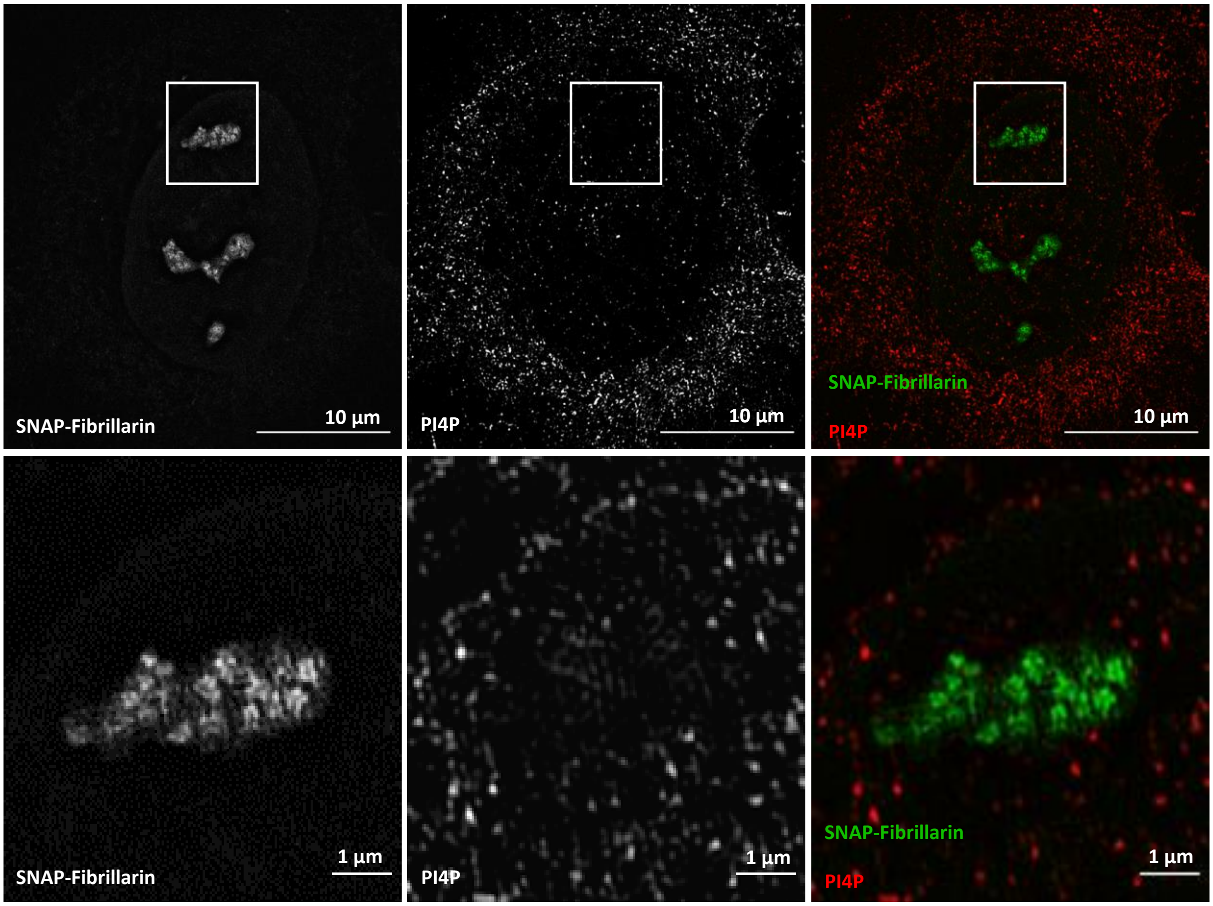
SNAP-Fibrillarin
10 µm
PI4P
10 µm
SNAP-Fibrillarin
PI4P
10 µm
SNAP-Fibrillarin
1 µm
PI4P
1 µm
SNAP-Fibrillarin
PI4P
1 µm

Supplementary figure 8. SIM images of Snap-Fibrillarin, PA andPI4P during interphase. A) SNAP-fibrillarin cell line fix and probed with spo20 to localize PA or in B) with anti-PI4P antibody. The inset of the figures show is magnified below. The fluorescent substrate used for this work was SNAP-Cell® Oregon Green®. Specific primary anti-PIP2 from Echelon™ (Z-A045) was used for immunofluorescence detection of PIP2 and labeled by anti-mouse IgM secondary antibody conjugated with Alexa Flour® 555 from Life Sciences. The PA sensor Spo20p-GFP was described previously [39].

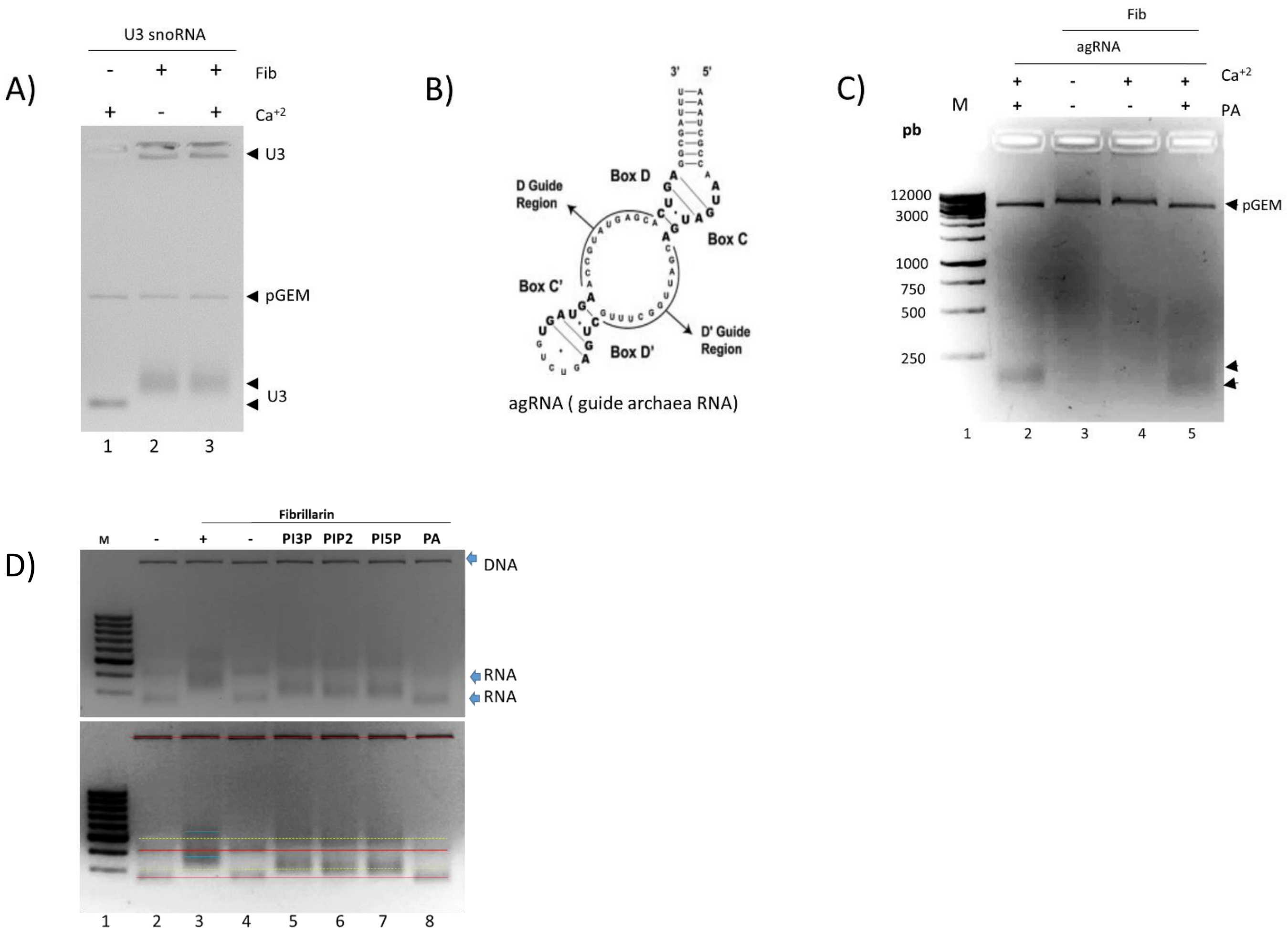

Supplementary figure 9. GMSA of fibrillarin in complex with snoRNA guides. Shift mobility gel assay of U3 snoRNA guide was evaluated with the presence of fibrillarin in (A) and another snoRNA from Archaea (C). The secondary structure of archaeal snoRNA guide is depicted in (B). Mobility shifts, highlighted by the red and dashed yellow lines, are observed in our *in vitro* assays in presence of three distinct phosphoinositides (D), implying that conformational changes could occur in presence of RNA, lipids and fibrillarin, lanes 5 to 7 in regard to lane 2 and 3.

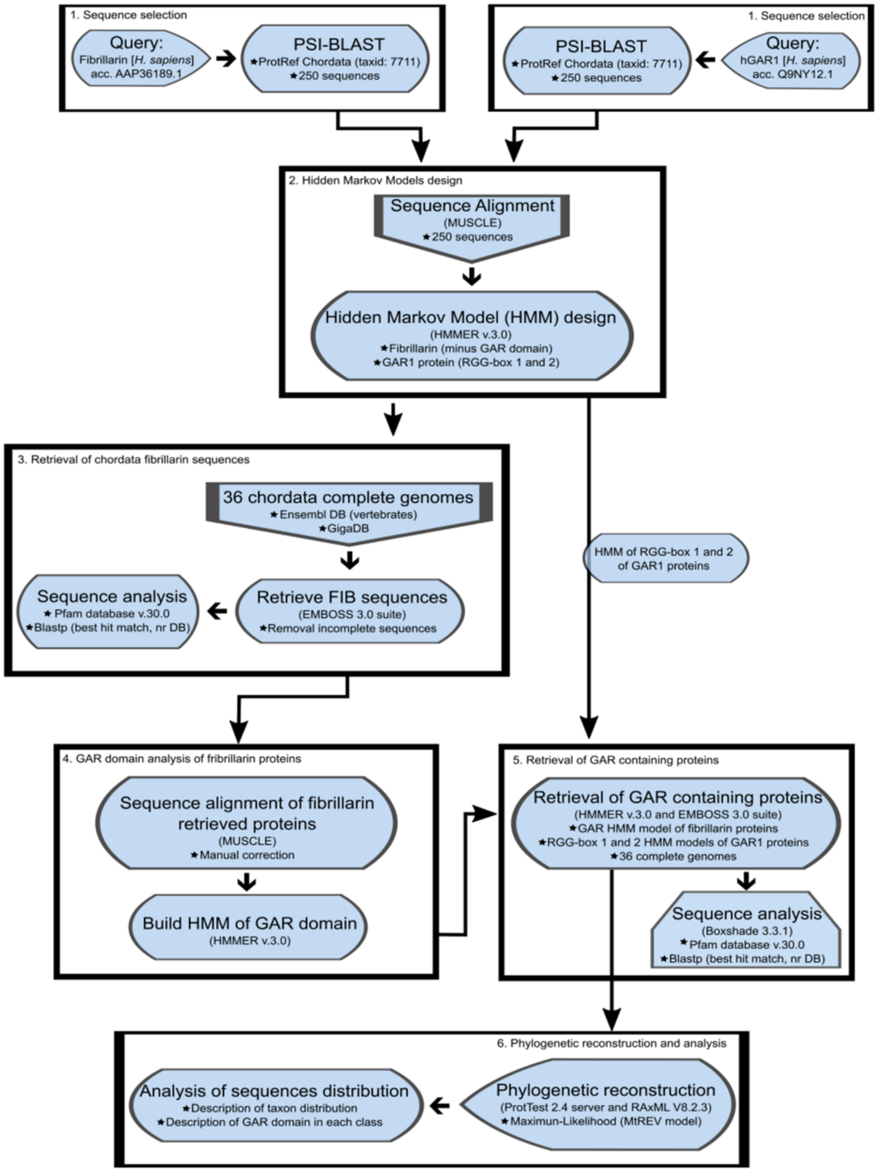

Supplementary figure 10. Flow diagram of sequences analyzed by bioinformatics tools. Bioinformatic analysis of the different sequences and domains evaluated in the work are presented as a flow chart with the different strategies used.

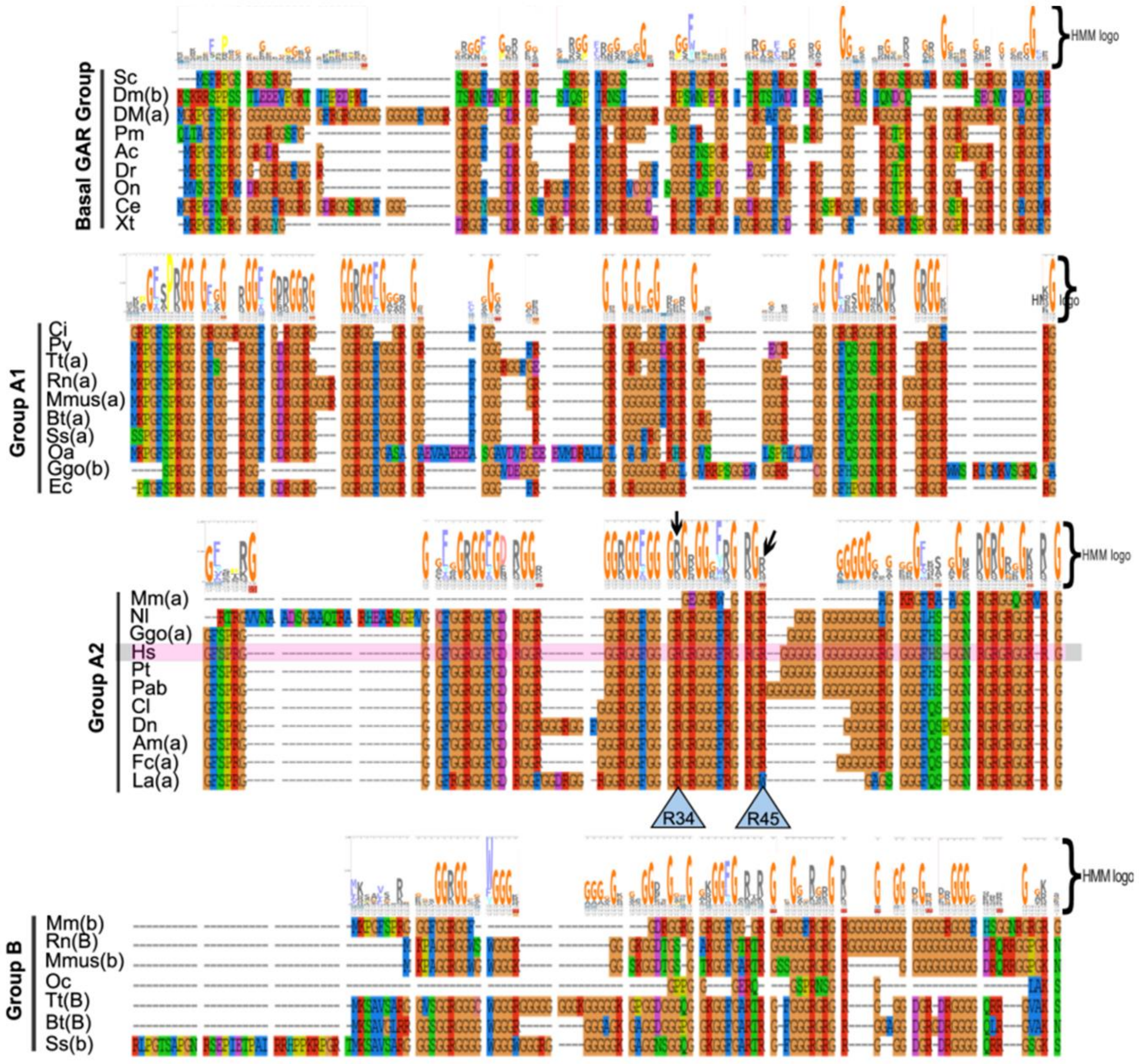

Supplementary figure 11. GAR domain amino acid alignment. The sequence alignment of the basal GAR group, group A1, group A2 and group B showing two conserved arginine: R34 and R45 are represented in the figure.

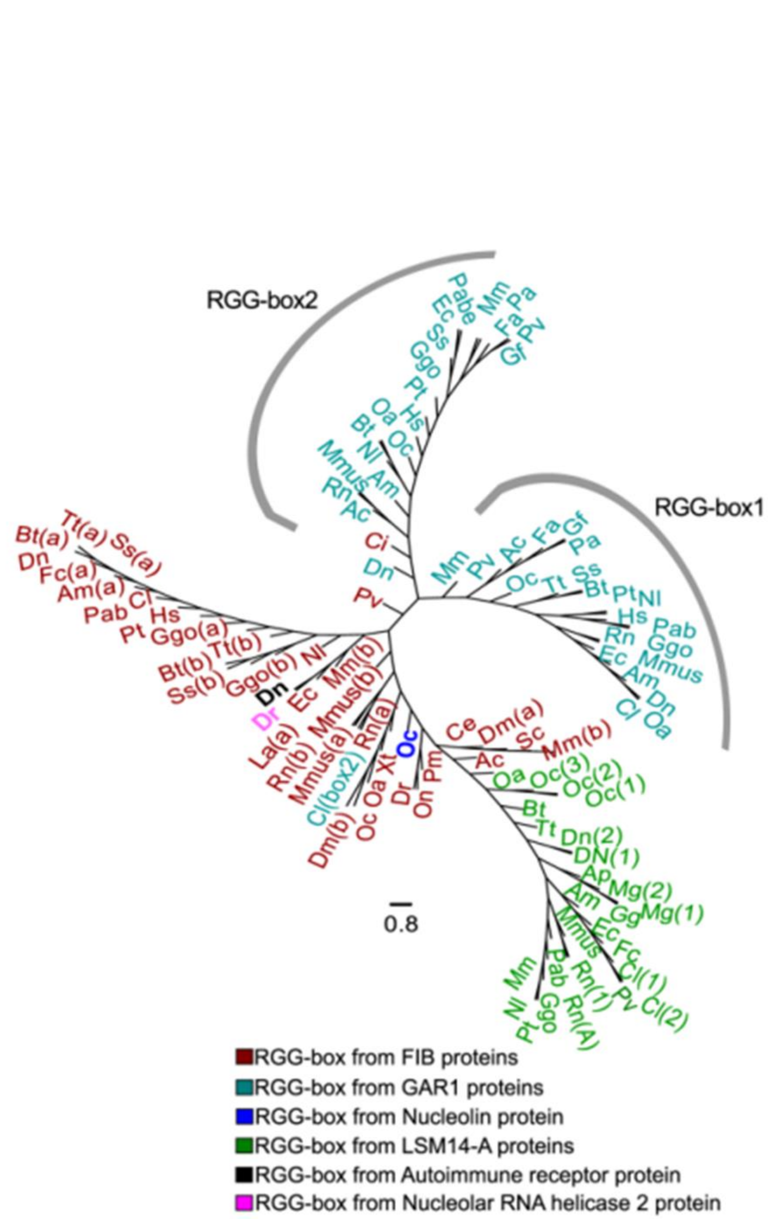

Supplementary figure 12. Phylogenetic analysis of the GAR domain branches founded in the proteomes of different species. Diagram showing the two branches from the HMM analysis of GAR1 protein sequences indicated by RGG-box1 and RGG-box2.

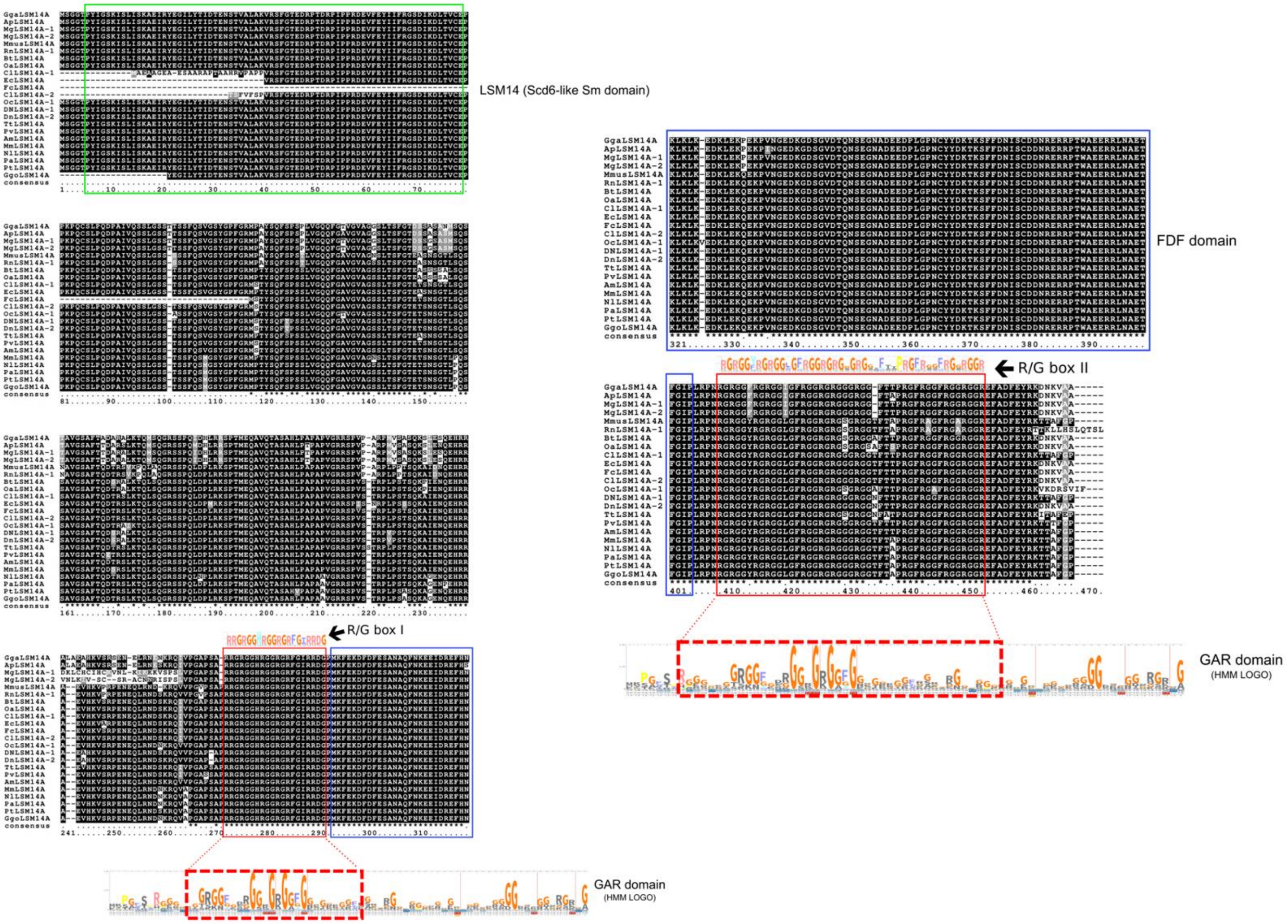

Supplementary figure 13. Boxshade of the Lms14-A protein sequences. The figure shows two distinct GAR boxes in the Lsm14-A sequences analyzed, indicated by arrows. The alignment consists in 23 LSM14-A proteins of vertebrate species containing two R/G rich regions characteristic of the non-conserved GAR domain structure of some nucleoli proteins. Green square corresponds to the LSM14 domain (Scd-like Sm domain), blue square corresponds to the FDF domain, red squares correspond to the GAR domain, and Red dashed square correspond to the section of GAR domain of fibrillarin from vertebrate species. The HMM-logo of GAR domain was constructed from the alignment of the GAR domains from the FIB retrieved proteins in chordate species.

Table S1. List of chordate species and thier proteome files used in the analyses of GAR domain (Fibrillarin and GAR1 proteins).
Date of retrieved proteomes: July 28th, 2016.

Specie	Database	Assembly	Proteome ID
Armadillo (*Dasypus novemcinctus*)	ensembl.org	Dasnov3.0	Dasypus_novemcinctus.Dasnov3.0.pep.all.fa
Cat (*Felis catus*)	ensembl.org	Felis_catus_6.2	Felis_catus.Felis_catus_6.2.pep.all.fa
Caenorhabditis elegans	ensembl.org	Wbcel235	Caenorhabditis_elegans.Wbcel235.pep.all.fa
Chicken (*Gallus gallus*)	ensembl.org	Galgal4	Gallus_gallus.Galgal4.pep.all.fa
Chimpanzee (*Pan troglodytes*)	ensembl.org	CHIMP2.1.4	Pan_troglodytes.CHIMP2.1.4.pep.all.fa
Ciona intestinalis	ensembl.org	KH	Ciona_intestinalis.KH.pep.all.fa
Cow (*Bos taurus*)	ensembl.org	UMD3.1	Bos_taurus.UMD3.1.pep.all.fa
Darwins finch (*Geospiza fortis*)	gigadb.org	Pygoscelis_adeliae	Geospiza_fortis.gene.pep
Dog (*Canis lupus familiaris*)	ensembl.org	CanFam3.1	Canis_familiaris.CanFam3.1.pep.all.fa
Dolphin (*Tursiops truncatus*)	ensembl.org	turTru1	Tursiops_truncatus.turTru1.pep.all.fa
Duck (*Anas platyrhynchos*)	ensembl.org	BGI_duck_1.0	Anas_platyrhynchos.BGI_duck_1.0.pep.all.fa
Elephant (*Loxodonta africana*)	ensembl.org	Loxafr3.0	Loxodonta_africana.loxAfr3.pep.all.fa
Flycatcher (*Ficedula albicollis*)	ensembl.org	FicAlb_1.4	Ficedula_albicollis.FicAlb_1.4.pep.all.fa
Fruitfly (*Drosophila melanogaster*)	ensembl.org	BDGP6	Drosophila_melanogaster.BDGP6.pep.all.fa
Gibbon (*Nomascus leucogenys*)	ensembl.org	Nleu1.0	Nomascus_leucogenys.Nleu1.0.pep.all.fa
Gorilla gorilla	ensembl.org	GorGor3.1	Gorilla_gorilla.gorGor3.1.pep.all.fa
Horse (*Equus caballus*)	ensembl.org	Equ Cab 2	Equus_caballus.EquCab2.pep.all.fa
Human (*Homo sapiens*)	ensembl.org	GRCh38.p7	Homo_sapiens.GRCh38.pep.all.fa
Lamprey (*Petromyzon marinus*)	ensembl.org	Pmarinus_7.0	Petromyzon_marinus.Pmarinus_7.0.pep.all.fa
Lizard (*Anolis carolinensis*)	ensembl.org	AnoCar2.0	Anolis_carolinensis.AnoCar2.0.pep.all.fa
Macaque (*Macaca mulatta*)	ensembl.org	MMUL 1.0	Macaca_mulatta.MMUL_1.pep.all.fa
Megabat (*Pteropus vampyrus*)	ensembl.org	pteVam1	Pteropus_vampyrus.pteVam1.pep.all.fa
Mouse (*Mus musculus*)	ensembl.org	GRCm38.p4	Mus_musculus.GRCm38.pep.all.fa
Orangutan (*Pongo abelii*)	ensembl.org	PPYG2	Pongo_abelii.PPYG2.pep.all.fa
Panda (*Ailuropoda melanoleuca*)	ensembl.org	ailMel1	Ailuropoda_melanoleuca.ailMel1.pep.all.fa
Penguin (Pygoscelis adeliae)	gigadb.org	Pygoscelis_adeliae	Pygoscelis_adeliae.pep
Pig (*Sus scrofa*)	ensembl.org	Sscrofa10.2	Sus_scrofa.Sscrofa10.2.pep.all.fa
Rabbit (*Oryctolagus cuniculus*)	ensembl.org	OryCun2.0	Oryctolagus_cuniculus.OryCun2.0.pep.all.fa
Rat (*Rattus norvegicus*)	ensembl.org	Rnor_6.0	Rattus_norvegicus.Rnor_6.0.pep.all.fa
Saccharomyces cerevisiae	ensembl.org	R64-1-1	Saccharomyces_cerevisiae.R64-1-1.pep.all.fa
Sheep (*Ovis aries*)	ensembl.org	Oar_v3.1	Ovis_aries.Oar_v3.1.pep.all.fa
Tilapia (*Oreochromis niloticus*)	ensembl.org	Orenil1.0	Oreochromis_niloticus.Orenil1.0.pep.all.fa
Turkey (*Meleagris gallopavo*)	ensembl.org	Turkey_2.01	Meleagris_gallopavo.UMD2.pep.all.fa
Xenopus tropicalis	ensembl.org	JGI 4.2	Xenopus_tropicalis.JGI_4.2.pep.all.fa
ZebraFinch (*Taeniopygia guttata*)	ensembl.org	TaeGut3.2.4	Taeniopygia_guttata.taeGut3.2.4.pep.all.fa
Zebrafish (*Danio rerio*)	ensembl.org	GRCz10	Danio_rerio.GRCz10.pep.all.fa

Table S2. List of fibrillarin protein sequeces retrieved from 36 complete genomes of vertebrate species and a yaest (S. cerevisiae).

*The FIB sequences were retrived from the listed genomes in S1 Table (a), using a HMM model build from an alignment of 250 FIB sequences recovered from the NCBI database by PSI-BLAST, against RefProteins of chordate species (taxid 7711) with 3 iterations.

**Specie**	**Seq_ID**	**BLASTp (best hit match against nr database)**
Armadillo (*Dasypus novemcinctus*)	ENSDNOP00000017037.2	PREDICTED: rRNA 2'-O-methyltransferase fibrillarin [Dasypus novemcinctus] XP_004449635.1
Cat (*Felis catus*)	ENSFCAP00000002787.3	PREDICTED: rRNA 2'-O-methyltransferase fibrillarin [Felis catus] XP_003997852.1
Cat (*Felis catus*)	ENSFCAP00000006575.3	PREDICTED: rRNA/tRNA 2'-O-methyltransferase fibrillarin-like protein 1 [Acinonyx jubatus]XP_014921259.1
Caenorhabditis elegans	T01C3.7.1	rRNA 2'-O-methyltransferase fibrillarin [Caenorhabditis elegans] NP_506691.1
Chimpanzee (*Pan troglodytes*)	ENSPTRP00000018825.2	Homo sapiens fibrillarin [synthetic construct] AAP36189.1
Ciona intestinalis	ENSCINP00000010410.3	PREDICTED: rRNA 2'-O-methyltransferase fibrillarin [Ciona intestinalis] XP_002131217.1
Cow (*Bos taurus*)	ENSBTAP00000003340.5	FBL protein [Bos taurus] AAI48118.1
Cow (*Bos taurus*)	ENSBTAP00000054113.1	TPA: rRNA/tRNA 2-O-methyltransferase fibrillarin-like protein 1-like [Bos taurus] DAA27197.1
Dog (*Canis lupus familiaris*)	ENSCAFP00000008079.3	PREDICTED: rRNA 2'-O-methyltransferase fibrillarin [Canis lupus familiaris] XP_013975919.1
Dolphin (*Ttursiops truncatus*)	ENSTTRP00000003736.1	PREDICTED: rRNA 2'-O-methyltransferase fibrillarin [Orcinus orca] XP_004271339.1
Dolphin (*Ttursiops truncatus*)	ENSTTRP00000015312.1	PREDICTED: rRNA/tRNA 2'-O-methyltransferase fibrillarin-like protein 1-like [Tursiops truncatus] XP_004315148.1
Elephant (*Loxodonta africana*)	ENSLAFP00000021738.1	PREDICTED: rRNA 2'-O-methyltransferase fibrillarin [Loxodonta africana] XP_010598699.1
Elephant (*Loxodonta africana*) (withou A	ENSLAFP00000021536.1	PREDICTED: LOW QUALITY PROTEIN: rRNA/tRNA 2'-O-methyltransferase fibrillarin-like protein 1 [Loxodonta africana] XP_010592483.1
Fruitfly (*Drosophila melanogaster*)	FBpp0071892	fibrillarin [Drosophila melanogaster] NP_523817.1
Fruitfly (*Drosophila melanogaster*)	FBpp0082153	uncharacterized protein Dmel_CG10909 [Drosophila melanogaster] NP_650236.1
Gibbon (*Nomascus leucogenys*)	ENSNLEP00000017336.2	FBRL_HUMAN [Homo sapiens] AAC28913.1
Gorilla gorilla	ENSGGOP00000021166.1	PREDICTED: rRNA 2'-O-methyltransferase fibrillarin [Gorilla gorilla gorilla] XP_004060777.1
Gorilla gorilla	ENSGGOP00000023049.1	PREDICTED: rRNA 2'-O-methyltransferase fibrillarin [Rhinopithecus roxellana] XP_010379187.1
Horse (*Equus caballus*)	ENSECAP00000020790.1	PREDICTED: rRNA 2'-O-methyltransferase fibrillarin isoform X2 [Equus przewalskii] XP_008530305.1
Human (*Homo sapiens*)	ENSP00000221801.2 (Chr19)	Homo sapiens fibrillarin [synthetic construct] AAP36189.1
Lamprey (*Petromyzon marinus*)	ENSPMAP00000008558.1	PREDICTED: rRNA 2'-O-methyltransferase fibrillarin [Pygocentrus nattereri] XP_017569907.1
Lizard (*Anolis carolinensis*)	ENSACAP00000016519.3	PREDICTED: rRNA 2'-O-methyltransferase fibrillarin [Anolis carolinensis] XP_003224982.1
Macaque (*Macaca mulatta*)	ENSMMUP00000029923.2	PREDICTED: rRNA 2'-O-methyltransferase fibrillarin [Balaenoptera acutorostrata scammoni] XP_007180034.1
Macaque (*Macaca mulatta*)	ENSMMUP00000016439.2	hypothetical protein EGK_17111 [Macaca mulatta] EHH27014.1
Megabat (*Pteropus vampyrus*)	ENSPVAP00000010225.1	PREDICTED: rRNA 2'-O-methyltransferase fibrillarin [Pteropus alecto] XP_006905463.1
Mouse (*Mus musculus*)	ENSMUSP00000037613.6	rRNA 2'-O-methyltransferase fibrillarin [Mus musculus] NP_032017.2
Mouse (*Mus musculus*)	ENSMUSP00000128889.1	rRNA/tRNA 2'-O-methyltransferase fibrillarin-like protein 1 [Mus musculus] NP_001004147.1
Orangutan (*Pongo abelii*)	ENSPPYP00000011171.2	PREDICTED: rRNA 2'-O-methyltransferase fibrillarin isoform X1 [Pongo abelii] XP_002829265.1
Panda (*Ailuropoda melanoleuca*)	ENSAMEP00000013342.1	hypothetical protein PANDA_012965 [Ailuropoda melanoleuca] EFB16332.1
Panda (*Ailuropoda melanoleuca*)	ENSAMEP00000007468.1	PREDICTED: rRNA/tRNA 2'-O-methyltransferase fibrillarin-like protein 1 [Ailuropoda melanoleuca] XP_011222456.1
Pig (*Sus scrofa*)	ENSSSCP00000019383.1	PREDICTED: rRNA 2'-O-methyltransferase fibrillarin [Sus scrofa] XP_003355972.2
Pig (*Sus scrofa*)	ENSSSCP00000018025.2	PREDICTED: rRNA/tRNA 2'-O-methyltransferase fibrillarin-like protein 1 [Sus scrofa] XP_003134119.1
Rabbit (*Oryctolagus cuniculus*)	ENSOCUP00000006534.2	PREDICTED: rRNA/tRNA 2'-O-methyltransferase fibrillarin-like protein 1 [Oryctolagus cuniculus] XP_008253597.2
Rabbit (*Oryctolagus cuniculus*)	ENSOCUP00000007791.2	PREDICTED: LOW QUALITY PROTEIN: rRNA 2'-O-methyltransferase fibrillarin [Oryctolagus cuniculus] XP_008249747.2
Rat (*Rattus norvegicus*)	ENSRNOP00000026021.3	rRNA 2'-O-methyltransferase fibrillarin [Rattus norvegicus] NP_001020814.1
Rat (*Rattus norvegicus*)	ENSRNOP00000020554.6	rRNA/tRNA 2'-O-methyltransferase fibrillarin-like protein 1 [Rattus norvegicus] NP_001102294.1
Saccharomyces cerevisiae	YDL014W	rRNA methyltransferase NOP1 [Saccharomyces cerevisiae S288c] NP_010270.1
Sheep (*Ovis aries*)	ENSOARP00000006790.1	PREDICTED: rRNA 2'-O-methyltransferase fibrillarin isoform X1 [Camelus bactrianus] XP_010945543.1
Tilapia (*Oreochromis niloticus*)	ENSONIP00000010128.1	PREDICTED: rRNA 2'-O-methyltransferase fibrillarin [Oreochromis niloticus] XP_003446214.2
Xenopus tropicalis	ENSXETP00000033811.3	rRNA 2'-O-methyltransferase fibrillarin [Xenopus tropicalis] NP_989101.1
Zebrafish (*Danio rerio*)	ENSDARP00000070509.4	rRNA 2'-O-methyltransferase fibrillarin [Danio rerio] NP_998167.1

Table S3. List of sequence proteins containing a GAR domain structure, retrieved from 36 complete genomes species by a HMM model from the GAR domain of FIB proteins.

**Specie**	**Seq_ID**	**BLASTp (best hit match, nr database)**
Armadillo (*Dasypus novemcinctus*)	ENSDNOP00000023544.1	PREDICTED: LOW QUALITY PROTEIN: autoimmune regulator [Dasypus novemcinctus] XP_004475201.1
Rabbit (*Oryctolagus cuniculus*)	ENSOCUP00000021169.1	PREDICTED: nucleolin isoform X1 [Pteropus vampyrus] XP_011382009.1
Zebrafish (*Danio rerio*)	ENSDARP00000087581.5	nucleolar RNA helicase 2 [Danio rerio] NP_001120807.2
Armadillo (*Dasypus novemcinctus*)	ENSDNOP00000023628.1	PREDICTED: protein LSM14 homolog A isoform X1 [Dasypus novemcinctus] XP_004450334.1
Armadillo (*Dasypus novemcinctus*)	ENSDNOP00000017784.1	PREDICTED: protein LSM14 homolog A isoform X1 [Dasypus novemcinctus] XP_004450334.1
Cat (*Felis catus*)	ENSFCAP00000019008.1	PREDICTED: protein LSM14 homolog A [Acinonyx jubatus] XP_014928577.1
Chicken (*Gallus gallus*)	ENSGALP00000007884.3	protein LSM14 homolog A [Gallus gallus] NP_001012796.1
Chimpanzee (*Pan troglodytes*)	ENSPTRP00000018507.3	PREDICTED: protein LSM14 homolog A isoform X4 [Pan troglodytes] XP_003316307.1
Cow (*Bos taurus*)	ENSBTAP00000000831.4	PREDICTED: protein LSM14 homolog A isoform X2 [Bison bison bison] XP_010847251.1
Dog (*Canis lupus familiaris*)	ENSCAFP00000010900.3	PREDICTED: protein LSM14 homolog A isoform X1 [Canis lupus familiaris] XP_005616816.1
Dog (*Canis lupus familiaris*)	ENSCAFP00000032804.2	PREDICTED: protein LSM14 homolog A [Acinonyx jubatus] XP_014928577.1
Dolphin (*Tursiops truncatus*)	ENSTTRP00000012216.1	PREDICTED: protein LSM14 homolog A isoform 2 [Tursiops truncatus] XP_004310724.1
Duck (*Anas platyrhynchos*)	ENSAPLP00000009178.1	LSM14A mRNA processing body assembly factor [Anas platyrhynchos] NP_001297343.1
Gibbon (*Nomascus leucogenys*)	ENSNLEP00000014270.1	PREDICTED: protein LSM14 homolog A isoform X3 [Nomascus leucogenys] XP_003281323.1
Gorilla gorilla	ENSGGOP00000001736.2	protein LSM14 homolog A isoform a [Homo sapiens] NP_001107565.1
Horse (*Equus caballus*)	ENSECAP00000013997.1	PREDICTED: protein LSM14 homolog A [Equus asinus] XP_014693579.1
Macaque (*Macaca mulatta*)	ENSMMUP00000028469.2	protein LSM14 homolog A [Macaca mulatta] NP_001244883.1
Megabat (*Pteropus vampyrus*)	ENSPVAP00000014172.1	PREDICTED: protein LSM14 homolog A isoform X1 [Mustela putorius furo] XP_004765254.1
Mouse (*Mus musculus*)	ENSMUSP00000082723.5	protein LSM14 homolog A [Mus musculus] NP_080224.1
Orangutan (*Pongo abelii*)	ENSPPYP00000011009.2	PREDICTED: protein LSM14 homolog A isoform X2 [Pongo abelii] XP_009230677.1
Panda (*Ailuropoda melanoleuca*)	ENSAMEP00000017933.1	PREDICTED: protein LSM14 homolog A isoform X2 [Ailuropoda melanoleuca] XP_011230569.1
Rabbit (*Oryctolagus cuniculus*)	ENSOCUP00000025827.1	PREDICTED: protein LSM14 homolog A-like isoform X1 [Oryctolagus cuniculus] XP_002722116.1
Rabbit (*Oryctolagus cuniculus*)	ENSOCUP00000010203.2	PREDICTED: protein LSM14 homolog A-like isoform X1 [Oryctolagus cuniculus] XP_002722116.1
Rabbit (*Oryctolagus cuniculus*)	ENSOCUP00000022522.1	PREDICTED: protein LSM14 homolog A-like isoform X1 [Oryctolagus cuniculus] XP_002722116.1
Rat (*Rattus norvegicus*)	ENSRNOP00000069131.1	PREDICTED: protein LSM14 homolog A isoform X1 [Rattus norvegicus] XP_006228919.1
Rat (*Rattus norvegicus*)	ENSRNOP00000030800.4	PREDICTED: protein LSM14 homolog A isoform X1 [Rattus norvegicus] XP_006228919.1
Sheep (*Ovis aries*)	ENSOARP00000004788.1	PREDICTED: protein LSM14 homolog A isoform X2 [Ovis aries] XP_004015197.1
Turkey (*Meleagris gallopavo*)	ENSMGAP00000018068.1	PREDICTED: protein LSM14 homolog A isoform X2 [Gallus gallus] XP_015147928.1
Turkey (*Meleagris gallopavo*)	ENSMGAP00000006000.2	PREDICTED: protein LSM14 homolog A isoform X2 [Gallus gallus] XP_015147928.1
